# Effects of Antioxidant Amino Acids on Cancer Sarcopenia

**DOI:** 10.3390/ijms26010272

**Published:** 2024-12-31

**Authors:** Shota Nukaga, Rina Fujiwara-Tani, Takuya Mori, Isao Kawahara, Ryoichi Nishida, Yoshihiro Miyagawa, Kei Goto, Hitoshi Ohmori, Kiyomu Fujii, Takamitsu Sasaki, Chie Nakashima, Yi Luo, Shingo Kishi, Ruiko Ogata, Hiroki Kuniyasu

**Affiliations:** 1 Department of Molecular Pathology, Nara Medical University School of Medicine, Kashihara 634-8521, Japan; shota.nukaga@gmail.com (S.N.); mori.takuya.n98@kyoto-u.jp (T.M.); isao_kawahara@a011.broada.jp (I.K.); g.m__r1@outlook.jp (R.N.); y.miya1103@gmail.com (Y.M.); ilgfgtk@gmail.com (K.G.); brahmus73@hotmail.com (H.O.); toto1999-dreamtheater2006-sms@nifty.com (K.F.); takamitu@fc4.so-net.ne.jp (T.S.); c-nakashima@naramed-u.ac.jp (C.N.); lynantong@hotmail.com (Y.L.); nmu6429@yahoo.co.jp (S.K.); pkuma.og824@gmail.com (R.O.); 2 Division of Rehabilitation, Hanna Central Hospital, Ikoma 630-0243, Japan; 3 Department of Medical Ethics and Genetics, Kyoto University, Kyoto 606-8501, Japan; 4 Osaka International Cancer Institute, Osaka 540-0008, Japan; 5 Department of Pathological Diagnosis, Nozaki Tokushukai Hospital, Daito 574-0074, Japan

**Keywords:** cancer sarcopenia, cystine, theanine, BCAA, metabolism

## Abstract

Cancer sarcopenia is highly prevalent in patients with advanced cancer, which is closely related to the disease prognosis. Overcoming cancer sarcopenia is important for cancer treatment. Cystine and theanine (CT), antioxidant amino acids, have been applied to the nutritional intervention of various diseases but their effects remain unclear on cancer sarcopenia. We attempt to examine the effect of CT on cancer sarcopenia. Both mouse and in vitro cachexia models showed that CT reduced oxidative stress, inhibited autophagy and apoptosis, improved oxidative phosphorylation and the suppression of high mobility group box-1 production, and improved sarcopenia and muscle maturity. When treated with 5-fluorouracil in a mouse cachexia model, tumor weight decreased but oxidative stress increased and muscle weight and muscle maturity were suppressed regardless of diet. However, in the CT group, oxidative stress was reduced and the exacerbation of sarcopenia by 5-fluorouracil was suppressed. Thus, in cancer cachexia, oxidative stress plays a major role in skeletal muscle damage, and CT, which has an anti-oxidative stress effect, has a strong protective effect on skeletal muscle. In the future, it will be important to conduct clinical studies on nutritional intervention for cancer sarcopenia using CT.

## 1. Introduction

Sarcopenia is defined as a state of decreased skeletal muscle mass and muscle strength or physical function that occurs in old age and refers to a state of decreased muscle function. In 2016, it was granted the International Classification of Diseases (ICD-10) code M62.84 and was changed from being classified as a syndrome to being classified as a disease [1]. In response to this, the Japanese Society on Sarcopenia and Frailty published sarcopenia treatment guidelines in Japan in 2017 [2]. According to this study, sarcopenia is present in 6–12% of elderly people in Japan and is also frequently present in 11–74% of patients with malignant diseases [2].

Cancer cachexia occurs in 40% of patients with all stages of cancer [3,4] and is even more common in the elderly and patients with advanced cancer [5]. Cachexia is a metabolic syndrome characterized by muscle loss [6,7], and skeletal muscle atrophy is considered the primary etiology of cachexia [8]. In addition, it is recognized that chemotherapy and certain targeted agents can cause sarcopenia and reduce physical function and quality of life [9]. Importantly, in cancer patients, skeletal muscle mass is associated with treatment tolerance [10] and is strongly positively correlated with survival [11].

Emphasized factors in cancer sarcopenia include cancer-related inflammatory cytokines [12], increased oxidative stress [13], increased catabolism [14], and anorexia [10]. Analysis using a mouse cachexia model has shown that increased oxidative stress and impaired mitochondrial energy metabolism are strongly involved [15]. It has been suggested that high mobility group box-1 (HMGB1), an inflammatory cytokine, leads to oxidative stress, inflammatory response, and autophagy in skeletal muscle [16].

The amino acid focused on in this study is important for skeletal muscle maintenance in cancer sarcopenia [17]. Inadequate protein intake is a key feature of cancer, and a broader range of proteins is recommended, but the specific components of amino acids that counteract muscle wasting have yet to be established [18]. Branched-chain amino acids play an important role in promoting protein synthesis in muscle tissue. In healthy older adults, high doses of leucine induce protein synthesis, but it is unclear whether long-term supplementation leads to increased muscle mass [19]. The effects of branched-chain amino acids on cancer are also expected to be notable, but further studies are awaited [20]. Experimental results have shown that glutamine increases lean body mass in postoperative patients [21], but larger studies are needed [20]. Glutamine and cysteine have antioxidant, anti-inflammatory, and immunomodulatory effects [22]. Cystine is the stable form of cysteine. Since dietary glutamate is metabolized during intestinal transit, oral administration of L-theanine, which is converted to glutamate in the liver, functions as a glutamate donor [23]. Glutathione is an important factor that neutralizes oxidative stress and maintains cellular redox and is associated with longevity and has been shown to be effective in suppressing sarcopenia [24,25]. Cystine and theanine (CT) thus both promote the synthesis of glutathione, a potent antioxidant. The combination of both protects tissue from cellular damage due to oxidative stress [26]. There have been no reports to date that cystine or theanine is effective against sarcopenia. However, because the involvement of oxidative stress is considered important as a cause of sarcopenia [13], especially cancer-related sarcopenia, it is expected that the combined use of cystine and theanine, which may promote the maintenance of redox, will be effective against cancer-related sarcopenia.

Thus, despite strong interest in the effects of amino acids on skeletal muscle, the effects of CT in cancer cachexia have not been sufficiently investigated. In this study, we will compare the effects of CT on cancer sarcopenia using a mouse model of cancer cachexia that we have developed [15].

## 2. Results

### 2.1. Effects of CT on Skeletal Muscle in Non-Tumor-Bearing Conditions

**Figure 1:**
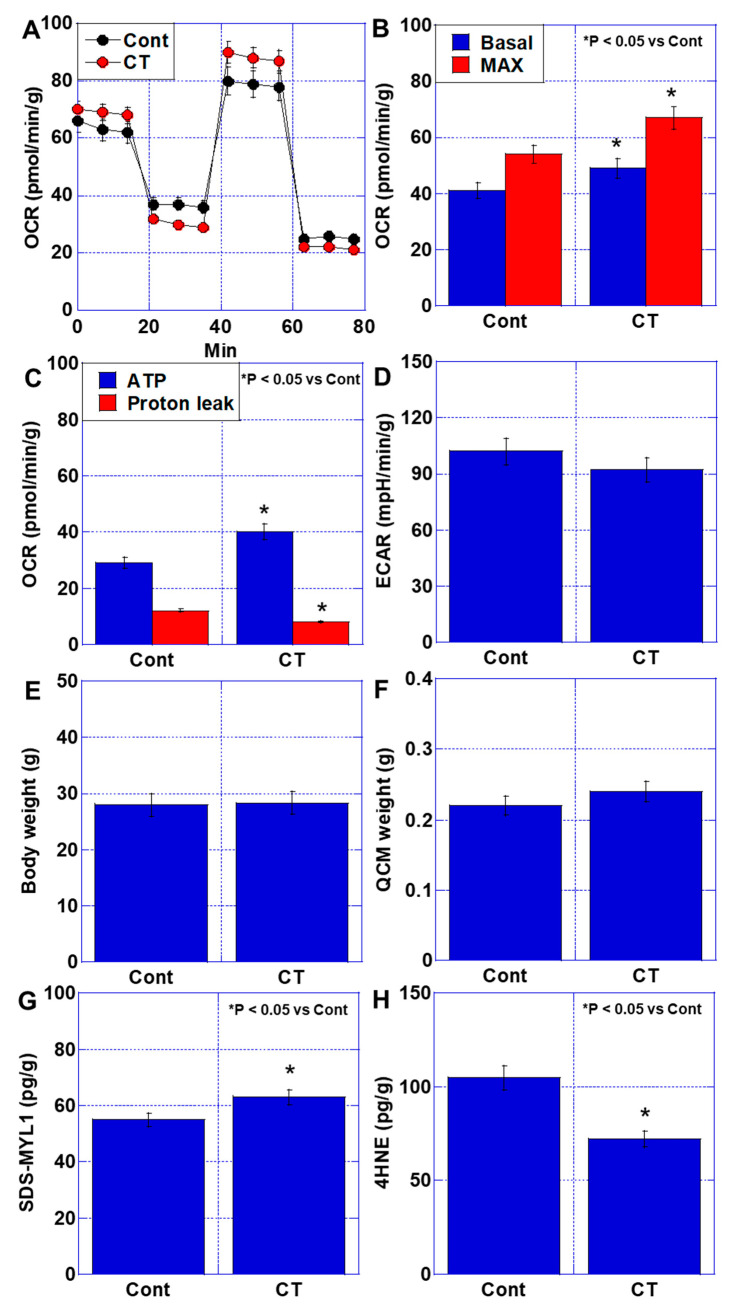
Effect of CT on skeletal muscle in a non-tumor-bearing condition. (**A**–**D**) Energy metabolism. (**A**) Oxidative phosphorylation by flux analysis. (**B**) Basal and maximum respiration. (**C**) ATP production and proton leak. (**D**) Glycolysis by ECAR. (**E**–**H**) Non-tumor-bearing mouse model fed with control diet (Cont) or CT diet for 2 weeks. (**E**) Body weight. (**F**) QCM weight. (**G**) Muscle maturity by SDS-soluble myosin light chain-1 (SDZ-MYL1). (**H**) Muscle oxidative stress by 4-hydroxynonenal (4HNE). Error bar: standard deviation from three independent trials or five mice. Statistical differences were calculated by ordinary ANOVA test. Basal—basal respiration; Cont—control; CT—cystine and theanine; ECAR—extracellular acidification rate; MAX—maximum respiration; QCM—quadriceps muscle; OCR—oxygen consumption rates.

First, we cultured C2C12 cells in normal medium and examined the effects of CT, which has antioxidant effects, on energy metabolism (Figure 1A–D). Basal and maximum respirations and ATP were increased by CT treatment (Figure 1A,B). In contrast, proton leak was decreased (Figure 1C). The extracellular acidification rate (ECAR) was not different between the control and CT treatment (Figure 1D). Non-tumor-bearing mice were administered CT diet for 2 weeks (Figure 1E–H). There was no significant difference in body weight and quadriceps femoris muscle (QCM) weight between the CD and CT groups (Figure 1E,F). However, sodium dodecyl sulfate-soluble myosin light chain-1 (SDS-MYL1), which is a marker for muscle maturation [15], increased more in the CT group than that in CD group (Figure 1G). In contrast, oxidative stress (4-hydroxynonenal, 4HNE) decreased in the CT group (Figure 1H). These findings suggest that CT has a skeletal muscle protecting effect in non-tumor-bearing conditions.

### 2.2. Effect of CT on Cancer Sarcopenia

**Figure 2:**
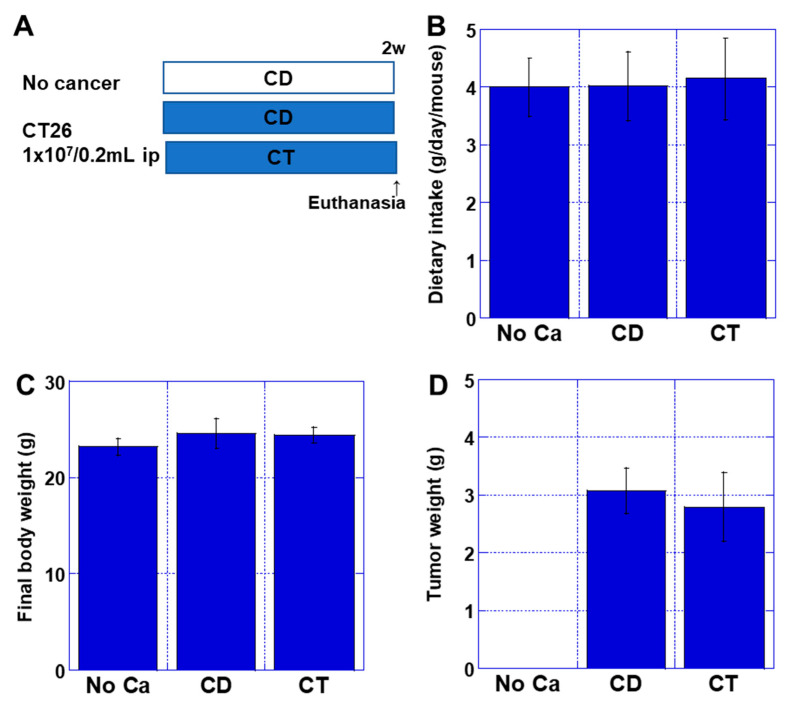
Effect of CT on cancer cachexia. (**A**) An experimental protocol. Mice were fed with CE-2 standard diet (No cancer {No Ca} group and CD group), and CT diet (Table 1, CT group). Each group employed five mice. (**B**) Dietary intake. (**C**) Body weight. (**D**) Tumor weight. Error bar: standard deviation from five mice. Statistical differences were calculated by ordinary ANOVA test. CD—control diet; CT—cystine and theanine; No Ca—no cancer.

We then compared the effects of CT in cancer cachexia. The mouse cachexia model used a system in which mouse colon cancer cells CT26 were intraperitoneally inoculated into syngeneic BALB/c mice [15]. The non-tumor inoculation group was administered a standard diet (No Ca group). The tumor inoculation group was divided into two groups: mice fed with control diet (CD group) and with CT diet (CT group) (Figure 2A). There were no significant differences between groups in food intake, body weight at euthanasia, and tumor weight (Figure 2B–D).

**Figure 3:**
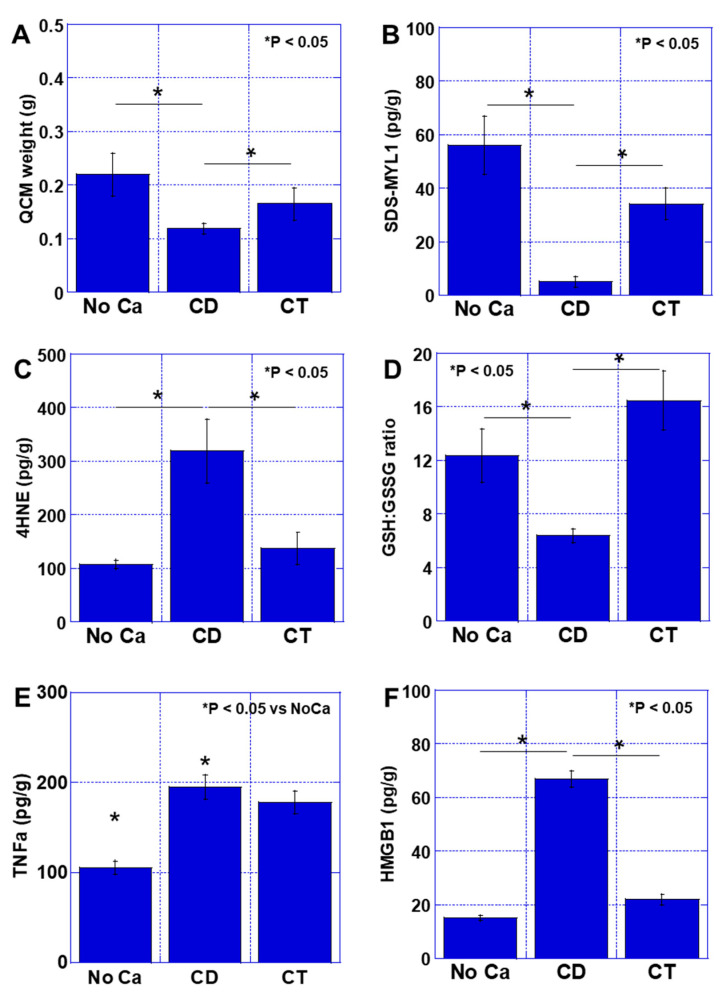
Effect of CT on cancer sarcopenia. Mice were fed with CE-2 standard diet (No cancer {No Ca} group and CD group), and CT diet (Table 1, CT group). Each group employed five mice. (**A**) QCM weight. (**B**) Muscle maturity by SDS-soluble myosin light chain-1 (SDS-MYL1). (**C**) Muscle oxidative stress by 4-hydroxynonenal (4HNE). (**D**) Muscle redox by GSH/GSSG ratio. (**E**,**F**) Intramuscular cytokines: TNFα (**E**) and HMGB1 (**F**). Error bar: standard deviation from five mice. Statistical differences were calculated by ordinary ANOVA test. CD—control diet; CT—cystine and theanine; GSH—glutathione; GSSG—glutathione disulfide; HMGB1—high mobility group box-1; No ca—no cancer; QCM—quadriceps muscle; TNF—tumor necrosis factor.

The weight of QCM decreased by 49% in the CD group compared to the No Ca group (Figure 3A). In contrast, in the CT group, the QCM weight loss was only 22%, which was significantly higher than the CD group. Furthermore, the SDS-MYL1 content decreased to 6 pg/g in the CD group compared to 56 pg/g in the No Ca group (Figure 3B). In contrast, in the CT group, SDS-MYL1 was 37 pg/g, significantly higher than in the CD group. Additionally, 4HNE was significantly higher in the CD group (296% of the No Ca group), whereas it decreased to the same levels of the No Ca group in the CT group (Figure 3C). The glutathione/glutathione disulfide (GSH:GSSG) ratio was significantly lower in the CD group than those in the No Ca and CT groups (Figure 3D). Tumor necrosis factor (TNF)-α and high group mobility box-1 (HMGB1) are known to be involved in cachexia [14]. The intramuscular concentration of TNFα was higher in the CD group than the No Ca group. There was no significant change in the concentration of TNFα in the CT group compared to that in the CD group (Figure 3E). Intramuscular HMGB1 was more increased in the CD group than that in the No Ca group; however, HMGB1 was significantly lower in the CT group than in the CD group (Figure 3F). Thus, CT showed a muscle protective effect in the mouse cancer sarcopenia model.

### 2.3. Effects of CT on Cancer-Related Impairment of Energy Metabolism

**Figure 4:**
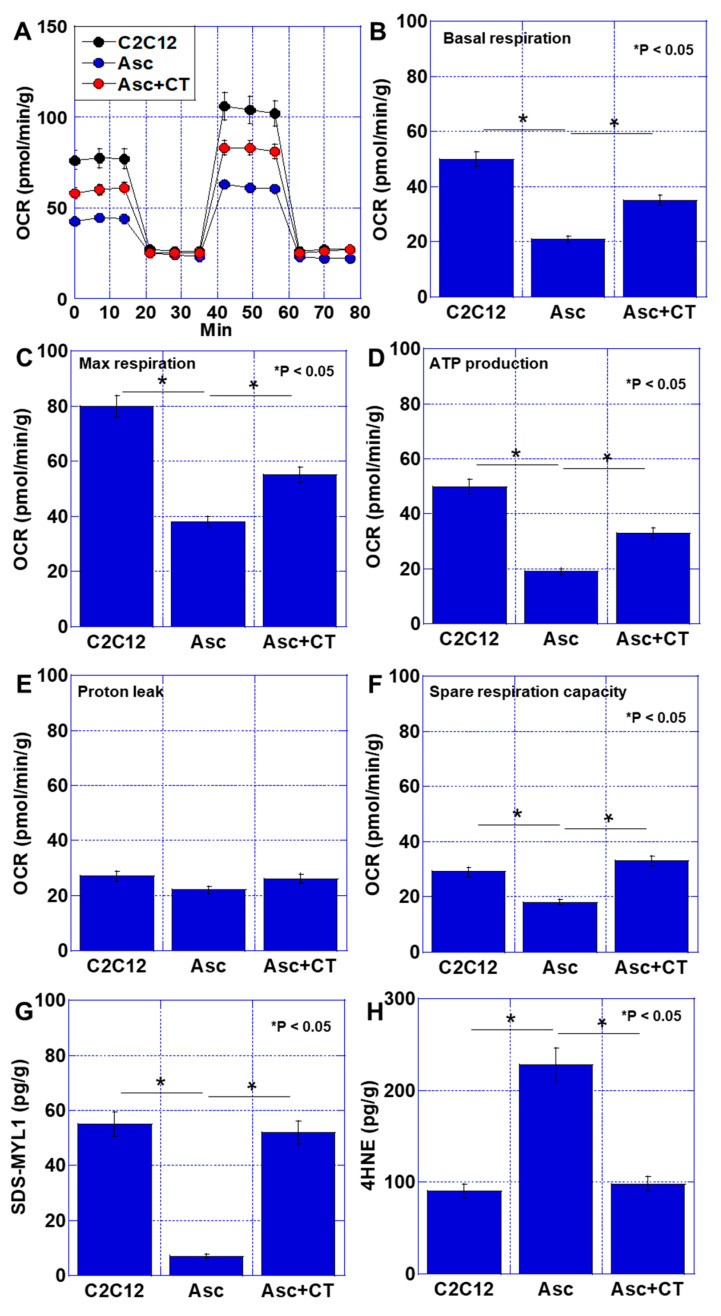
Effect of CT on energy metabolism in an in vitro cachexia model. (**A**) Oxidative phosphorylation by flux analysis. (**B**) Basal respiration. (**C**) Maximum respiration. (**D**) ATP production. (**E**) Proton leak. (**F**) Spare respiration capacity. (**G**) Muscle maturity by SDS-soluble myosin light chain-1 (SDS-MYL1). (**H**) Muscle oxidative stress by 4-hydroxynonenal (4HNE). Error bar: standard deviation from three independent trials. Statistical differences were calculated by ordinary ANOVA test. Asc—cancer ascites; C2C12—untreated control; CT—cystine and theanine; OCR—oxygen consumption rates.

Next, an in vitro cachexia model, in which mouse myoblast C2C12 is treated with the ascites of the mouse cachexia model described in our previous report [19], was treated with CT, and the effects on mitochondrial energy metabolism were compared (Figure 4). Ascites treatment reduced basal respiration, maximum respiration, and ATP production to less than 1/2 of those in the control C2C12 cells (Figure 4A–D). In contrast, ascites+CT treatment reduced basal respiration, maximum respiration, and ATP production to 2/3 of the control C2C12 cells; however, in Asc+CT treatment, significant recovery was observed compared to ascites treatment alone (Figure 4A–D). There were no significant changes in proton leak among all treatments (Figure 4E). Spare respiratory capacity was decreased in ascites treatment, but it recovered in ascites+CT treatment (Figure 4F). SDS-MYL1 was decreased in ascites treatment compared with control C2C12 cells, but recovered in ascites+CT treatment (Figure 4G). Additionally, 4HNE was increased in ascites treatment compared with C2C12, but recovered in ascites+CT treatment (Figure 4F). Thus, CT recovered the impairment of oxidative phosphorylation by cancer ascites.

### 2.4. Effect of CT on Apoptosis

**Figure 5:**
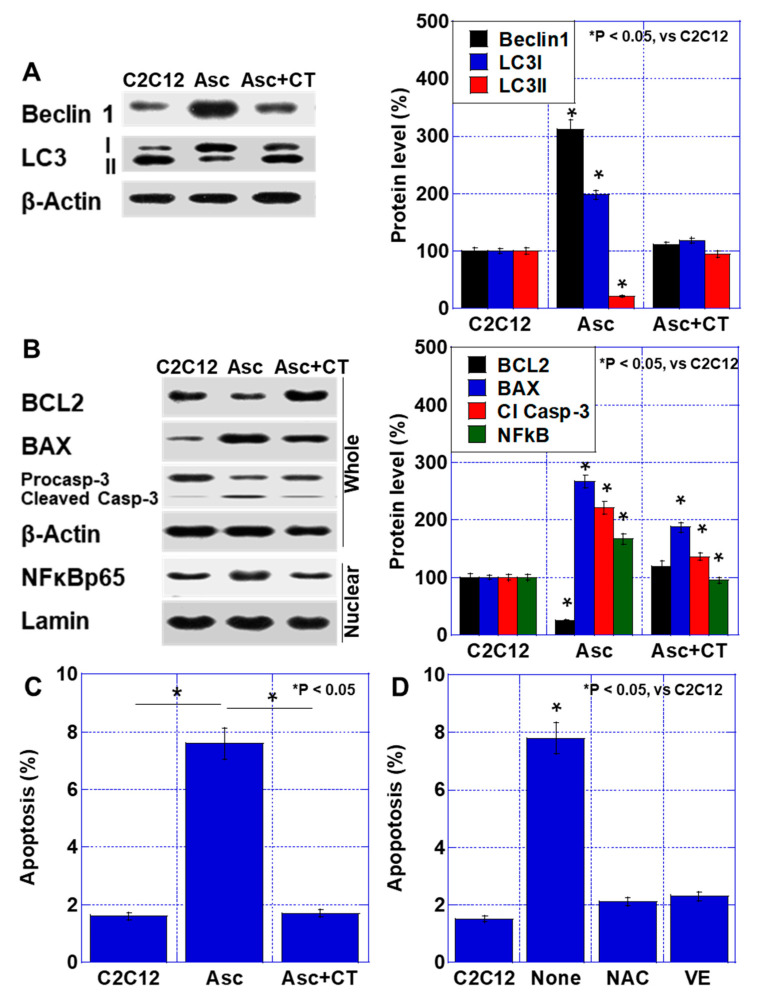
Effect of CT on apoptosis. (**A**) Levels of autophagy-associated proteins. Right panel: semi-quantification of Western blot. (**B**) Levels of apoptosis-associated proteins. Right panel: semi-quantification of Western blot. (**C**) Apoptotic cells. (**D**) Effect of antioxidants on apoptosis. NAC (1 mM) or VE (20 μM) were treated for 48 h. Error bar: standard deviation from three independent trials. Statistical differences were calculated by ordinary ANOVA test. Asc—cancer ascites; BAX—Bcl2-associated X protein; BCL2—B-cell lymphoma-2; C2C12—untreated control; CT—cystine and theanine; LC—microtubule-associated protein light chain 3; NAC—N-acetyl-L-cysteine; NF—nuclear factor; Nuclear—nuclear fraction; VE—vitamin E; Whole—whole cell lysate.

Next, we investigated the effects of CT on apoptosis in C2C12 cells using an in vitro cachexia model (Figure 5A). Ascites treatment increased Beclin 1, microtubule-associated protein light chain 3 (LC3)-I, and the LC3I/II ratio, which are autophagy executing proteins. Such changes were suppressed by CT (Figure 5A). Next, we examined changes in apoptosis-related proteins by ascites treatment and found that B-cell lymphoma-2 (BCL2) decreased, Bcl2-associated X protein (BAX) increased, and cleaved caspase-3 appeared. Also, nuclear factor (NF)-κBp65 was increased in nuclear fraction. In contrast, CT partially recovered such changes (Figure 5B). Apoptotic cells were increased by ascites treatment but recovered to the control level by CT (Figure 5C). Ascites-induced apoptosis was also suppressed by N-acetyl cysteine (NAC) or vitamin E (VE). These findings suggest that CT suppresses muscular apoptosis by its antioxidant effect (Figure 5D).

### 2.5. Effect of CT When Combined with 5-Fluorouracil (5FU) Treatment in a Mouse Model of Cachexia

**Figure 6:**
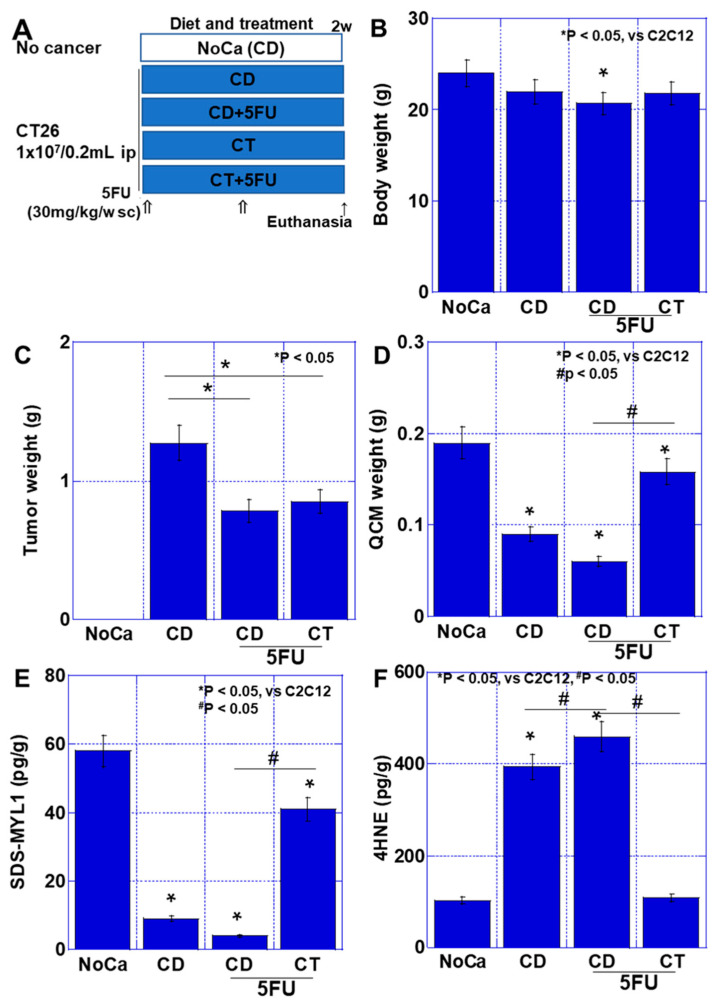
Effect of CT on cancer sarcopenia in 5FU-administered mice. (**A**) An experimental protocol. CT26 mouse colon cancer cells (1 × 10^7^) were inoculated into the peritoneal cavity (ip) of syngeneic BALB/c mice (5-week-old, male). Then, 5FU (30 mg/kg/week) was administered subcutaneously (sc) once a week. Mice were divided into five groups, with five mice each: No Ca group (CD diet, no CT26 cancer cells and no 5FU), CD group (CD diet, CT26 cells ip, no 5FU), CD+5FU group (CD diet, CT26 cells ip and 5FU+), CT group (CT diet [Table 1], CT26 cells ip, no 5FU), and CT+5FU group (CT diet, CT26 cells ip and 5FU+). (**B**) Body weight at euthanasia. (**C**) Tumor weight. (**D**) QCM weight. (**E**) Muscle maturity by SDS-soluble myosin light chain-1 (SDZ-MYL1). (**F**) Muscle oxidative stress by 4-hydroxynonenal (4HNE). Error bar: standard deviation from five mice. Statistical differences were calculated by ordinary ANOVA test. 5FU—5-fluorouracil; CD—control diet (CF-2 diet); CT—cystine and theanine; QCM—quadriceps muscle.

It is known that chemotherapy worsens cancer sarcopenia [9,27]. Finally, we examined sarcopenia during cancer chemotherapy and the effect of CT on it in the mouse cachexia models (Figure 6). For this, 5FU (30 mg/kg), which is equivalent to the IC50 of CT26 cells, was administered on a mouse cachexia model fed with CT diet (Figure 6A). In body weight, the CT+5FU group showed lower weight than the no tumor control mice (No Ca group) (Figure 6B). Tumor weight was reduced by 5FU treatment in the CD group and also in the CT group. It was found that 5FU reduced tumor weight by 35–40% in both groups, but there was no significant difference between the CD and CT groups (Figure 6C). QCM weights were lower in all groups of tumor-bearing mice than in the no cancer group. In addition, 5FU reduced QCM weight in the CD+5FU and CT+5FU groups. Even with 5FU, the QCM weight in the CT+5FU group was 2.2 times that of the CD+5FU group, and exacerbation of sarcopenia due to 5FU was suppressed (Figure 6D). Furthermore, when SDS-MYL1 was examined, it was lower in all groups than in the No Ca group. In addition, 5FU administration decreased SDS-MYL1 in the CD+5FU and CT+5FU groups. However, in the CT+5FU group, SDS-MYL1 was 10-fold higher than in the CD+5FU group. Thus, CT also inhibited the exacerbation of 5FU-related muscle maturity impairment (Figure 6E). In muscle tissue, 4HNE increased 3.9-fold in the CD group compared to the No Ca group. In the CD+5FU group, 4HNE was increased 4.6-fold compared to the No Ca group, whereas the CT+5FU group showed 4HNE levels that remained the same as the No Ca group. Thus, CT recovered 5FU-induced oxidative stress in the muscle.

## 3. Discussion

Examining the effects of CT on cancer sarcopenia, CT reduced skeletal muscle volume and muscle maturity by reducing oxidative stress, suppressing autophagy and apoptosis, improving oxidative phosphorylation, and suppressing HMGB1 production.

We employed an in vitro cachexia model using mouse cancer ascites, which contained TNFα and HMGB1. In ascites treatment, NFkB activation, BCL2 decrease, and BAX increase were observed and apoptosis was enhanced. CT showed the normalization of NFkB activation, BCL2, and BAX, and apoptosis that had been enhanced by ascites treatment recovered to normal levels. CT supplies substrates for GSH production, cysteine and glutamate, and promotes GSH synthesis [28]. In our mouse experiments, the antioxidant effect of CT was clearly observed. CT showed the suppression of 4HNE and improvement of the GSH:GSSG ratio. The antioxidant effect of CT has been reported. The combination of CT protects tissue from cellular damage caused by free radicals [26]. For skeletal muscle, C2C12 myotubes have been reported to improve maximal mitochondrial respiration, which is reduced by oxidative stress [22,29]. CT has been reported to reduce radiation enteritis by suppressing oxidative stress [30]. CT induces GSH production and suppresses autophagy, NFκB activation, and apoptosis [31]. Regulation of oxidative stress by GSH contributes to the suppression of apoptosis [32], as oxidative stress plays an important role in the induction of autophagy and apoptosis. In our study, antioxidants such as NAC and VE also showed anti-apoptotic effects. Thus, the antioxidant effect of CT is thought to be responsible for the muscle protecting effect of CT.

CT showed no suppression of TNFα expression, but markedly decreased HMGB1 expression. It has been reported that CT suppresses perioperative and inflammation-associated increases in interleukin (IL)-6 and increases anti-inflammatory IL-10 [28]. Thus, CT works to regulate the cytokine response via the GSH redox cycle [33]. The HMGB1 inhibitory effect of CT that we discovered is also considered to be one of the anti-inflammatory effects of CT. HMGB1 is a cytokine that induces autophagy in skeletal muscle [12], is increased in the blood of cancer patients, and plays an important role in the development of sarcopenia [16]. In this respect as well, CT is highly useful in improving cancer sarcopenia. Furthermore, HMGB1 acts as an autocrine/paracrine growth factor in many cancers, enhancing malignant phenotypes and disease recurrence [34], and the suppression of HMGB1 by CT is thought to play a role in maintaining antitumor effects during cancer chemotherapy.

Treatment with 5FU decreased tumor weight in both diet groups, but increased oxidative stress and suppressed QCM weight and muscle maturity. In the CT group, 5FU-induced exacerbation of sarcopenia was suppressed along with the reduction in oxidative stress. This result indicates that anticancer drug treatment exacerbates sarcopenia through the induction of oxidative stress. At the same time, we have shown that CT, which has an antioxidant effect, suppresses the exacerbation of sarcopenia without reducing the antitumor effect of anticancer drugs. CT has been reported to reduce the side effects of oxaliplatin, capecitabine, S-1, and 5FU, but does not affect antitumor efficacy [26,35]. The mechanism of the CT effect is that the tissue oxidative stress caused by anticancer drugs is counteracted by the induction of GSH by CT [26,35]. In ascites-treated conditions, although oxidative phosphorylation and glycolysis were promoted, the increase in ATP production was found in CT. CT increases in QCM weight and muscle maturity under normal conditions. In contrast, in the tumor-bearing condition, CT showed relevant protective effects to skeletal muscle.

Our study showed that CT was effective against cancer-related sarcopenia. However, this result was only in mice and did not take into account species differences in dosage and metabolism. Furthermore, this was the result of short-term administration, and the long-term effects are unclear. In the future, extensive studies using various dosages and different animal species are necessary. At the same time, it is essential to examine its usefulness in humans.

## 4. Materials and Methods

### 4.1. Cell Culture

The CT26 mouse colon cancer cell line was a kind gift from Professor Isaiah Josh Fidler (MD Anderson Cancer Center, Houston, TX, USA) [36]. The C2C12 mouse myoblasts were purchased from Dainihon Pharmacy Co. (Tokyo, Japan). Cells were cultured in Dulbecco’s modified Eagle’s medium (DMEM; Wako Pure Chemical Industries, Ltd., Osaka, Japan) supplemented with 10% fetal bovine serum (Sigma-Aldrich Chemical Co., St. Louis, MO, USA).

### 4.2. In Vitro Cachexia Model

For the in vitro cachexia model [37], supplemented regular medium was combined with ascites of CT26 cell-inoculated BALB/c mice at 20% *v*/*v*. For the control, supplemented regular medium was combined with a cultured medium of CT26 cells at 20% *v*/*v*. The comparison of components of the fresh DMEM, ascites-added DMEM, and cultured medium-added DMEM is shown in Table 1.

### 4.3. Animals

Five-week-old male BALB/c mice were purchased from SLC Japan (Shizuoka, Japan). The animals were kept in a pathogen-free animal facility under a 12/12 h light/dark cycle in a temperature (22 °C)- and humidity-controlled environment, in accordance with the institutional guidelines approved by the Committee for Animal Experimentation of Nara Medical University, Kashihara, Japan, following current regulations and standards of the Japanese Ministry of Health, Labor and Welfare (approval no. 12777, 20 April 2020). Animals were acclimated to their housing for seven days before the start of the experiment.

The mouse cachexia model was based on previous reports of intraperitoneal inoculation of CT26 cancer cells into syngeneic BALB/c mice [15]. For treatment with 5FU (5-fluorouracil), 5FU (30 mg/kg body weight) was injected subcutaneously once a week. The preparation of skeletal muscles was conducted according to our previous report [15]; the quadriceps femoris muscle (QCM) was separated from the bones.

### 4.4. Diet

A CE-2 diet (CLEA Japan, Inc., Tokyo, Japan) was used as the control diet and a CT diet was prepared by mixing cystine and theanine with the control diet, as shown in Table 2. Intakes of food and calories per mouse were calculated based on the total daily intake of 5 mice in each group.

### 4.5. Protein Extraction

Protein was extracted from cells according to our previous report [37]. Whole-cell lysates were prepared in a 0.1% SDS-added RIPA-buffer (Thermo Fisher Scientific, Tokyo, Japan). The Minute Cytoplasmic and Nuclear Extraction Kit (Invent, Biotechnologies, Inc., Plymouth, MN, USA) was used to extract the nuclear fraction. Protein assay was performed using a Protein Assay Rapid Kit (Wako Pure Chemical Corporation, Osaka, Japan).

### 4.6. Immunoblot Analysis

Lysates were separated using 10% sodium dodecyl sulfate (SDS)-polyacrylamide gel electrophoresis and transferred onto nitrocellulose membranes. The membranes were then incubated with primary antibodies (Table 3) followed by peroxidase-conjugated IgG antibodies (P0217; Dako, Glostrup, Danish). Immune complexes were visualized using the Fusion Solo imaging system (M&S Instruments Inc., Osaka, Japan).

### 4.7. Enzyme-Linked Immunosorbent Assay (ELISA)

Whole-cell lysates and mitochondrial fraction were prepared as described above. Protein assays were performed using a Protein Assay Rapid Kit (Wako Pure Chemical Corporation, Osaka, Japan). The ELISA kits used are listed in Table 3. ELISA was performed according to the manufacturer’s instructions.

### 4.8. Mitochondrial Stress Test (Seahorse Assay)

The mitochondrial stress test and glycolytic stress test were performed as described in our previous report [19]. The oxygen consumption rates (OCR) and ECAR of 1 × 10^4^ viable C2C12 cells per well were measured using the Seahorse XFe24 Extracellular Flux Analyzer with Seahorse XF24 FluxPaks (Agilent Technologies, Chicopee, Kitchener, ON, Canada).

### 4.9. Glycolytic Stress Test

The ECAR of C2C12 cells was measured using a modified glycolytic stress test in the Seahorse XFe24 Extracellular Flux Analyzer with Seahorse XF24 FluxPaks (Agilent Technologies, Chicopee, Kitchener, ON, Canada). C2C12 cells were cultured in a growth medium in 6-well plates with the ascites or the cultured medium before Seahorse experiments. GIC cells (1 × 10^4^ cells/well) were later plated in the XF base medium (Agilent Technologies, Chicopee, Canada) containing 200 mM L glutamine and 5 mM HEPES, as recommended by the manufacturer for glycolytic assays. The sensor cartridge apparatus was rehydrated one day in advance by adding 1 mL XF Calibrant to each well and incubating at 37 °C until needed. The injection ports of the sensor cartridge apparatus were loaded with the following drugs, in a chronological order of four injections, to meet the indicated final concentrations in the wells: 10 mM glucose, 1 µM oligomycin, 1 µM rotenone, and 5 µM antimycin A and 50 mM 2-deoxyglucose (combined injection). Treatment with the rotenone/antimycin combination allowed an assessment of the impact of electron transport on ECAR by respiratory acidification coupled to the passage of some glycolytic pyruvate through the TCA cycle to supply respiration.

### 4.10. Statistical Analysis

Because our experimental design included multiple, and sometimes three, data groups, we used ordinary ANOVA tests to compare the mean values of each group and determine whether there were significant differences using InStat software (version 3.1; GraphPad Software, Inc., La Jolla, CA, USA). Data are expressed as the mean ± standard deviation of 3 independent experiments or 5 mice. *p* < 0.05 (two-sided) was considered to indicate statistical significance.

## 5. Conclusions

Our data show that amelioration of oxidative stress is useful in cancer sarcopenia. The CT used in this study induces GSH production, suggesting that redox balance improvement may have a more important effect than simply the suppression of oxidative stress. Regarding CT, their supplements are already commercially available, and clinical trials for cancer patients are desired in the future.

## Figures and Tables

**Table 1:** Component of media for treatment.

Component	Medium		
	D-MEM	Ascites Added ^(1)^	CM Added ^(2)^
Glucose (mg/dL)	450 ± 2	365 ± 4	385 ± 7
Pyruvate (mg/dL)	11 ± 0.1	9 ± 1	9 ± 1
Glutamine (mg/dL)	58 ± 0.2	51 ± 3	49 ± 3
Lactate (pmol)	0	6.4 ± 1.1	1.3 ± 0.3
HMGB1 (μg/mL)	ND	15 ± 0.9	ND
TNFα (pg/mL)	ND	11 ± 0.1	ND

^(1)^ Ascites was collected from mice 2 weeks after inoculation of CT26 mouse colon cancer cells (1 × 10^7^) intraperitoneally. Ascites was added at 20% *v*/*v* to fresh D-MEM culture medium supplemented with 10% FBS. ^(2)^ Cultured medium (CM) of CT26 cells (1 × 10^8^) was added at 20% *v*/*v* for 48 h to fresh D-MEM culture medium supplemented with 10% FBS. D-MEM—Dulbecco’s modified eagle medium; HMGB1—high mobility group box-1; TNFα—tumor necrosis factor-α; ND, not detected.

**Table 2:** Diet ingredients.

Ingredient	Control Diet	CT Diet
Moisture (%)	8.83	8.83
Crude protein (%)	25.13	25.13
Crude fat (%)	4.92	4.92
Crude fiber (%)	4.42	4.42
Crude ash (%)	6.86	6.86
NFE (%)	49.84	49.84
Valine (%)	-	-
Laucine (%)	-	-
Isoleucine (%)	-	-
Cystine (%)	-	0.161
L-Theanine (%)	-	0.064
Energy (kcal)	334.2	345.1

CT—cysteine and theanine; NFE—nitrogen-free extract.

**Table 3:** Antibodies and ELISA kits.

Target	Cat. No.	Company	Address
Antibodies			
Beclin1	ab92389	Abcam	Cambridge, MA, USA
Casapase-3	ab184787	Abcam	Cambridge, MA, USA
LC3	CTB-LC3-1-50	Cosmo Bio	Tokyo, Japan
NFκBp65	13629-1-AP	Proteintech	Rosemont, IL, USA
Lamin B1	66095-1-IG	Proteintech	Rosemont, IL, USA
BCL2	sc-7382	Santa-Cruz	Dallas, TX, USA
BAX	sc-7480	Santa-Cruz	Dallas, TX, USA
β-Actin	sc-47778	Santa-Cruz	Dallas, TX, USA
ELISA kit			
MYL1	CSB-EL015305MO	Cusabio Biotech	Houston, TX, USA
HMGB1	326078738	Shino Test	Sagamihara, Japan
Mouse TNFα	MTA00B	R&D Systems	Minneapolis, MN, USA
GSH:GSSG	CB-P050-K	Creative Biolabs	Shirley, NY, USA

NF—nuclear factor; LC3—microtubule-associated protein light chain 3; BCL2—B-cell lymphoma-2; BAX—BCL2-associated X protein; MYL1—myosine light chain-1; HMGB1—high mobility group box-1; TNF—tumor necrosis factor; GSH—glutathione; GSSG—glutathione disulfide.

## Data Availability

Data are contained within the article.

## Abbreviations

CT—cystine and theanine; HMGB1—high mobility group box-1; ECAR—extracellular acidification rate; QCM—quadriceps femoris muscle; SDS-MYL1—sodium dodecyl sulfate-soluble myosin light chain-1; 4HNE—4-hydroxynonenal; TNF—tumor necrosis factor; LC3—microtubule-associated protein light chain 3; BCL2—B-cell lymphoma-2; BAX—Bcl-2-associated X protein; NF—nuclear factor; 5FU—5-fluorouracil; GSH—glutathione; GSSG—glutathione disulfide; IL—interleukin.
